# Improved HDL Cholesterol through Vitamin D Status Correction Substantially Lowers 10-Year Atherosclerotic Cardiovascular Disease Risk Score in Vitamin D-Deficient Arab Adults

**DOI:** 10.3390/nu15030551

**Published:** 2023-01-20

**Authors:** Shaun Sabico, Kaiser Wani, William B. Grant, Nasser M. Al-Daghri

**Affiliations:** 1 Biochemistry Department, College of Science, King Saud University, Riyadh 11451, Saudi Arabia; 2 Sunlight, Nutrition and Health Research Center, P.O. Box 641603, San Francisco, CA 94164-1603, USA

**Keywords:** vitamin D correction, cardiovascular disease, ASCVD risk score, lipids, sex specific, HDL cholesterol

## Abstract

This interventional study aimed to determine whether correcting vitamin D status in deficient Arab adults [25(OH)D <50 nmol/L] improves their 10-year risk of Atherosclerotic Cardiovascular Disease (ASCVD) risk scores. Saudi adults (58 males 62 females) with baseline vitamin D deficiency (<50 nmol/L) were given 50,000 IU cholecalciferol weekly for 2 months, then twice a month, followed by daily 1000 IU until month 6. Fasting blood samples were collected pre- and post-intervention and assessed for glucose, lipids, and 25(OH)D levels. The predicted 10-year ASCVD risk scores were calculated at baseline and after intervention. At baseline, significantly higher 10-year ASCDV risk scores were observed in males than females (9% vs. 3%, *p* < 0.001). After 6 months, only 21% (25 out of 120) achieved 25(OH)D levels above optimal level (≥75 nmol/L). While modest improvements were seen in glucose and lipid profiles, only HDL cholesterol showed favorable significant changes in all participants, which translated to significantly improved 10-year ASCVD risk scores independent of whether they achieved optimum vitamin D status. Still, those who achieved optimal vitamin D levels had a modestly larger decrease in ASCVD risk scores than those with less optimal 25(OH)D levels (−23% versus −18%) and this improvement was slightly more pronounced in males (−26% versus −10%, or 16% improvement) than females (−47% versus −32%, or 15% improvement). In conclusion, vitamin D status correction significantly enhances HDL cholesterol which prospectively reduces 10-year ASCVD risk as vitamin D levels approach optimum status among adult Arabs with baseline vitamin D deficiency. This improvement appears to be slightly more apparent in males than females.

## 1. Introduction

The events related to cardiovascular diseases (CVDs), particularly ischemic heart disease (IHD) and stroke, are the leading causes of premature mortality and rising health care costs [1]. A recent review found that an estimated 18.6 million CVD-related deaths were reported worldwide in the year 2019 [2]. In the Middle East, and particularly in Saudi Arabia, IHD and stroke are currently ranked second and fourth, respectively, as the main causes of mortality, and these CVD events impose a significant financial burden on the health care system [3,4]. The primary risk factors for CVD events classically include obesity, hypertension, dyslipidemia, diabetes, smoking, poor diet, and physical inactivity [5,6]. The advent of rapid urbanization has led to a radical change in lifestyle in the last few decades in Saudi Arabia [7,8]. This unhealthy lifestyle change, which includes the adoption of Westernized dietary habits and a more sedentary lifestyle, had significantly increased the prevalence of chronic non-communicable diseases including CVD events in such a short time frame [9].

Patients at high risk of CVD are prioritized in preventive cardiology. Assessment of CVD risk begins with a global risk score, which is then used to determine the 10-year risk using conventional risk factors. The landmark Framingham Heart Study (FHS) observations documented for the first time the significance of early assessment of risk factors for an effective preventive CVD treatment [10]. Men and women were evaluated on separate scales, and calculated an individual’s 10-year risk of a CVD event based on age, total and HDL cholesterol, blood pressure, smoking, and diabetes status. In accordance with the recommendations of the third Adult Treatment Panel of the National Cholesterol Education Program (NCEP-ATP) in 2001 [11], this method was subsequently used to stratify individuals into low (10%), intermediate (10–20%) or high (>20%) risk profiles to determine who are more likely to benefit from intensive lifestyle modification in addition to drug therapy. Although many variants of the CVD risk assessment tool have been developed, the most recent guidelines from the American College of Cardiology and the American Heart Association (ACC/AHA) [12,13] recommend a tool called the Atherosclerotic Cardiovascular Disease (ASCVD) Risk Estimator Plus (tools.acc.org/ascvd-risk-estimator-plus) in people who do not already have CVD. This new score, such as the older ones, is based on a collection of established risk factors (Supplementary Figure S1) to estimate the likelihood of an ASCVD event occurring within the next decade.

Metabolomics has led to the discovery of novel molecules and biochemical pathways related to human diseases, including CVD [14,15]. Atherosclerotic lesions, for instance, are characterized as the accumulation of lipid molecules within the walls of large and medium arteries [16]. The molecular etiology involved in lipid metabolism may be key in understanding inflammatory processes in ASCVD. Our recent study identified at least 248 proteins exhibiting significant sexually dimorphic modulation in pathways associated with inflammation [17]. For instance, apolipoproteins were observed to be involved in pathways linking vitamin D function with lipid metabolism, suggesting that vitamin D levels influence levels of lipoproteins and, consequently, may modulate the risk of ASCVD events [18]. Vitamin D deficiency is defined as circulating levels of 25-hydroxy-vitamin D [25(OH)D] lower than 50 nmol/L, which is based on the recommendations of the Endocrine Society [19]. Recent studies have shown that vitamin D deficiency is a widespread issue all over the world [20].

Vitamin D, also called the “sunshine vitamin,” is best known for its role in calcium and skeletal health. Lately, however, vitamin D deficiency has also been strongly associated with extra-skeletal disorders, though the efficacy of vitamin D status correction given are still being debated given the variance in its protein receptors [21,22]. Several meta-analyses and systematic reviews suggest an association between vitamin D deficiency and chronic diseases such as obesity [23], metabolic syndrome [24], type 2 diabetes [25], and rheumatoid arthritis [26], inflammatory conditions which may progress to ASCVD events. Interestingly, the diseases mentioned are common among nations with a high prevalence of vitamin D deficiency, including Saudi Arabia and the entire Middle East and North Africa region, where established predictors of vitamin D deficiency include the female sex, culturally-appropriate clothing that requires full covering from head-to-toe, urbanization, excessive summer heat and low dietary calcium and vitamin D intake [27].

While recent interventional studies have shed light on decreasing the ASCVD risk score through lifestyle modification strategies [28,29], there is lack of prospective evidence specifically in the region on favorably altering ASCVD risk score through vitamin D supplementation. In order to fill this gap, the present study investigated whether vitamin D status correction among baseline vitamin D-deficient Arab adults confers favorable changes in their 10-year risk of ASCVD.

## 2. Materials and Methods

### 2.1. Participants and Intervention

The participants in this 6-month vitamin D interventional study included apparently healthy Saudi adults with established vitamin D deficiency [25(OH)D <50 nmol/L), randomly selected from the database of Chair for Biomarkers of Chronic Diseases (CBCD), as published earlier [18,30]. Participants who consented were clinically reassessed and those with type 2 diabetes, atherosclerosis, liver and kidney diseases, as well as those who reported use of vitamin D supplements at the time of recruitment were excluded from the study. A total of 120 male and female Saudi adults with baseline vitamin D deficiency were enrolled and provided with vitamin D supplements. The participants were advised to take 50,000 IU cholecalciferol (VitaD50000, Synergy Pharma, Dubai, UAE) weekly once for first two months, then once in two weeks for month 3 and 4, and a daily dose of 1000 IU (VitaD1000, Synergy Pharma, Dubai, UAE) for the last two months [31]. The study was conducted according to the guidelines of the Declaration of Helsinki and approved by the Ethics Committee of the College of Medicine, King Saud University, Riyadh, Saudi Arabia (Approval# E-15-1667).

### 2.2. Clinical Assessment, Samples and Biochemical Measurements

A standardized questionnaire was used by trained research personnel to collect demographic information, personal and family medical history and personal medication history. At baseline, the clinical assessment included information about age, whether or not the subject has been diagnosed with diabetes, family history of diabetes, CVD and dyslipidemia, and anthropometric assessment. For anthropometry, height (cm) and weight (kg) was assessed by standard weighing scale with an attached stadiometer. Waist (cm) and hip (cm) circumferences were assessed by standard tape measure. Systolic and diastolic blood pressures were measured in a sitting position twice using a standard procedure by the nurses that helped in the recruitment of participants with an automated BP instrument (Omron-705CP; Omron Corp., Tokyo, Japan) and the average value was obtained. Body mass index (BMI) was calculated by weight in kgs divided by square of height in meters.

Fasting blood samples were also collected two times, once prior to recruitment and another post supplementation. The samples collected were processed, aliquoted and transported to the CBCD laboratory and stored under appropriate storing conditions until analysis. The biochemical assessment included lipid profile and fasting glucose analyzed using commercially available assays on a routine biochemical analyzer, Konelab (Thermo-Fisher Scientific, Waltham, MA, USA). Serum 25(OH) D was measured on the COBAS e-411 automated analyzer (Roche Diagnostics, Indianapolis, IN, USA), using a commercially available assay (Elecsys^®^ Vitamin D total II, reference number 07464215). The assay has a measuring range of 7.5–250 nmol/L, a repeatability of less than 6%, and an intermediate precision of less than 7%, according to the manufacturer. CBCD has been a DEQAS (vitamin D External Quality Assessment Scheme) participating laboratory.

### 2.3. Predicted 10-Year Risk for ASCVD

The ASCVD Risk Estimator Plus is an online tool, based on a collection of established risk factors, to estimate the likelihood of an ASCVD event in the next 10 years in people who do not already have CVD. The risk factors included age, sex, systolic and diastolic blood pressures, circulating levels of total cholesterol, HDL cholesterol and LDL cholesterol, race, history of diabetes and smoking, and whether on statins, hypertension treatment, or aspirin [32]. The data collected from the participants at baseline and post supplementation was fed one at a time to the ASCVD Risk Estimator Plus and a current 10-year ASCVD risk score was generated for both time points. The scores obtained were categorized as low risk (<5%), borderline risk (5–7.4%), intermediate risk (7.5–19.9%), and high risk (≥20%).

### 2.4. Sample Size Calculation and Statistical Analysis

Sample size calculation was carried out based on data obtained from previous findings ascertaining the effects of 25(OH)D correction in HDL cholesterol levels [18,30]. G*Power was used to determine sample size given the effect size of 0.27 and power of 0.85. The minimum required total sample is N = 100 given α = 0.05.

All analyses were performed using statistical software SPSS version 23 (IBM, Chicago, IL, USA). For descriptive analysis, categorical variables were presented as frequency (percentages, %) and continuous variables were either presented as mean ± standard deviation (SD) or median (quartile 1, quartile 3) for normal and non-normal continuous variables, respectively. The data at baseline were divided into sex and the differences in these variables were tested using Chi-square tests for categorical variables, Mann–Whitney U tests for normal continuous variables, and Wilcoxon tests for non-normal continuous variables. The non-normal variables were log-transformed for normality before any further analysis. The average increase or decrease from baseline was presented for all variables by calculating post-supplementation values minus the baseline values and this change was tested for significance by paired *t*-test. The participants were then divided into those who achieved a post-supplementation vitamin D status of 75 nmol/L. This tertile stratification was carried out in the data for both sexes. Percentage (%) change for other biochemical parameters in this stratification was calculated by dividing the difference in average values post supplementation with baseline average values and multiplying by 100. Trend differences in percent changes in each variable in these two groups were calculated using analysis of variance (ANOVA). Changes in 10-year ASCVD risk scores based on this vitamin D improvement stratification were also calculated and presented. All the figures were plotted in Microsoft Excel 2016 and *p*-values < 0.05 were considered significant.

## 3. Results

### 3.1. Baseline Characteristics in the Participants

A total of 120 vitamin D-deficient Saudi adults with a mean age of 40.6 years were included in the study. Females were 51.7% (N = 62) of the total participants. A large proportion of the participants were either overweight (N = 59, 49%) or obese (N = 43, 36%). Diabetes was present in 22% (N = 27) of the study participants. Compared to females, males were significantly heavier (average of 83 vs 72 kgs, *p* < 0.01) and had higher systolic and diastolic blood pressures and moderately higher circulating levels of vitamin D at baseline (average of 34.7 vs 30.4 nmol/L, *p* = 0.03). Females, as expected, had higher hip circumferences compared to males. A higher baseline ASCVD risk score was observed in males than females (median levels of 9% vs 3%, *p* < 0.001). Table 1 presents the characteristics of the study participants at baseline.

**Figure 1:**
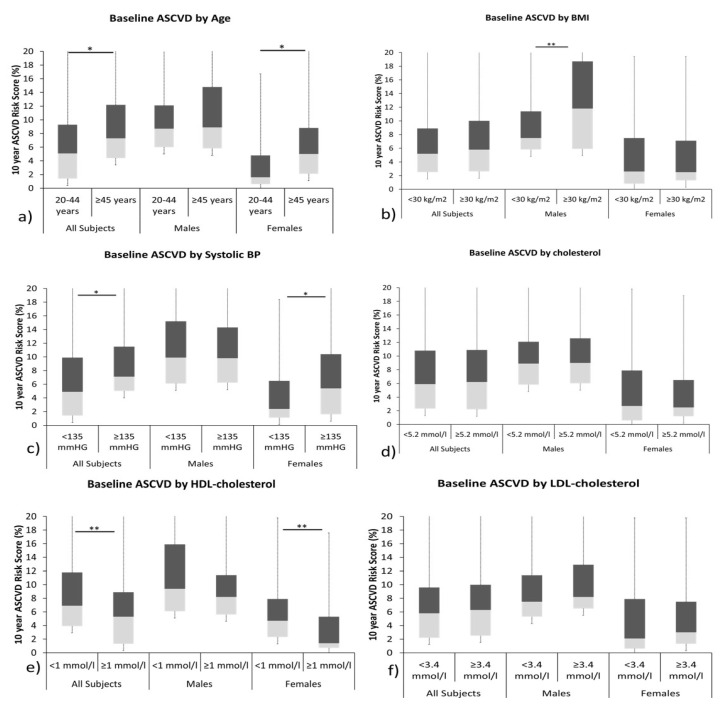
Box-plot representing ASCVD risk scores in study participants at recruitment, stratified according to (**a**) age, (**b**) BMI, (**c**) systolic blood pressure, (**d**) cholesterol levels, (**e**) HDL cholesterol, and (**f**) LDL cholesterol. * and ** statistical significance at *p* < 0.05 and *p* < 0.01 levels, respectively.

The baseline 10-year ASCVD risk scores for the study participants according to different risk factors were plotted as shown in Figure 1. Higher ASCDV risk scores were observed in males than females across all factors. BMI significantly affected the ASCVD risk scores in males while in females, it was significantly affected by age, systolic blood pressure, and HDL cholesterol. More females had low ASCVD risk scores (68%) and the majority of males (78%) had borderline to intermediate ASCVD risk scores.

### 3.2. Changes in Biochemical Characteristics Post Vitamin D Supplementation

Table 2 presents the changes in biochemical characteristics after 6 months. A significant increase in circulating 25(OH) D levels was observed in all participants. In males, 25(OH)D increased from baseline 34.7 nmol/L to 61.0 nmol/L (+76%, *p* < 0.001) while in females, it increased from baseline 30.4 nmol/L to 65.5 nmol/L (+116%, *p* < 0.001). In parallel, a significant increase in circulating HDL cholesterol levels were observed in both sexes. In males and females, respectively, an increase of 7% (*p* = 0.001) and 16% (*p* = 0.04) was observed post supplementation. Consequently, a significant decrease in average ASCVD scores was observed post vitamin D intervention. In males, average ASCVD risk scores decreased from 8.8 to 7.4 (a decrease of 16% compared to baseline ASCVD scores, *p* = 0.02) and similarly in females, average ASCVD risk scores decreased from 2.6 to 1.5 (a decrease of 42%, *p* < 0.001).

Post supplementation, none of the participants had severe vitamin D deficiency (<25 nmol/L) while 13% of females and 21% of males were within ≥ 25 nmol/L and <50 nmol/L, respectively (not shown in tables).

### 3.3. ASCVD Risk Scores as per Improvement in Circulating 25(OH) Vitamin D Levels Post Intervention

**Figure 2:**
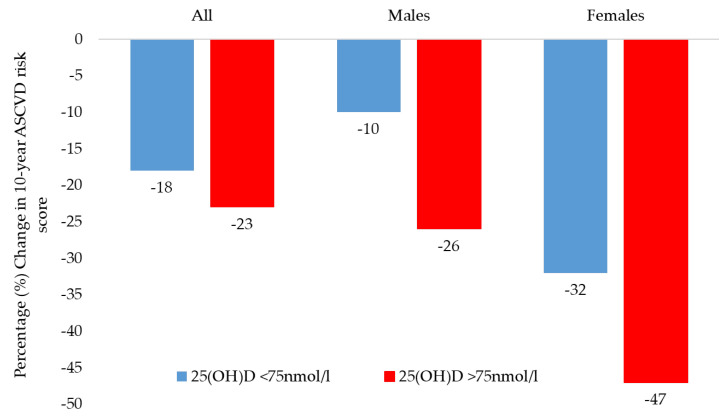
Percentage change in 10-year ASCVD risk score in participants who achieved 25(OH)D >75 nmol/L versus <75 nmol/L.

Table 3 shows the changes in glucose and lipid parameters with the corresponding changes in 10-year ASCVD risk scores in participants who achieved the target of 25(OH)D 75 nmol/L post supplementation. Only 25 out of 95 participants (21%; 9 males, 16 females) were able to achieve the target, corresponding to a mean percentage increase of 159%. No significant changes were seen in glucose and lipid profiles with the exception of HDL cholesterol which increased significantly post intervention in all groups as well as after stratification to sex. Compared to those who were unable to achieve the target, the reduction in ASCVD score was higher among those who achieved optimum levels, yet was not statistically significant (−23% versus −18%; *p* = 0.41). Stratification according to sex revealed the same non-significant but higher 10-year ASCVD risk score reductions in males and females (−26% versus −10%; *p* = 0.71 and −47% versus −32%; *p* = 0.69, respectively) (Figure 2). Stratification based on tertiles of vitamin D improvement is presented as Supplementary Table S1, wherein a significant overall trend of an increase in circulating HDL cholesterol levels was seen with more improvement in the three tertiles.

## 4. Discussion

The main finding in the present study is that among vitamin D-deficient Arab adults, vitamin D status correction modestly reduces ASCVD risk score. This improvement is mainly driven by the substantial increase in HDL cholesterol over time, which, from an ASCVD score perspective, is more favorable in females than males. The present finding echoes the abundance of evidence pointing to improved lipid profiles secondary to vitamin D supplementation [33], HDL cholesterol in particular [34]. The present study also confirms, based on several observational studies on Arab ethnic groups, that vitamin D supplementation may also be more advantageous in women in terms of its extra-skeletal effects, despite the higher cardiometabolic associations of vitamin D status in Arab males than females [35,36].

How vitamin D supplementation favorably affects HDL cholesterol may partly be explained by its influence in HDL’s fractional components, namely the apolipoproteins. Apolipoprotein A1, for instance, is a major structural and functional protein of HDL cholesterol, both of which are considered anti-atherogenic [37]. In a recent meta-analysis involving seven randomized controlled trials (RCTs) on vitamin D and apolipoprotein A, substantial increases in apolipoprotein A levels were noted only in RCTs with <12 weeks of supplementation duration (standardized mean difference, SMD 0.71 mg/dl; *p* = 0.04) and those receiving daily vitamin D supplementation (SMD 0.56 mg/dl, *p* = 0.03) [38]. Several vitamin D intervention studies in Saudi Arabia have also constantly shown this favorable increase in HDL cholesterol ranging from 12 to 18 months [39,40], although increases in other HDL components such as apolipoproteins C and E were observed overtime, instead of apolipoprotein A. These intervention studies, while not considered RCTs, comply with the criterion of temporality in Hill’s criteria for causality and should be considered acceptable despite inherent inferiority over RCTs [41,42]. Lastly, cross-sectional studies also constantly observed significant inverse associations of vitamin D status with other lipid profiles, with significant positive correlations only to HDL cholesterol [43,44].

While some observational studies suggest an inverse correlation between vitamin D status and CVD events, large randomized controlled trials (RCTs) have been unable to establish that supplementing with vitamin D can reduce the risk of CVD [45]. In order to evaluate whether or not vitamin D influences CVD outcomes, a new method known as Mendelian randomization (MR) research has been developed, in which genetically predicted 25(OH)D concentrations are determined by genome-wide association studies (GWAS) [46]. Non-linear MR analysis of data from 44,519 cases of CVD incidence and 251,269 controls from the UK Biobank recently suggested the causality of vitamin D in reducing the risk of CVD, and the results of the present study are consistent with these previous findings [47]. According to the results of the MR study, the CVD rates were noticeably elevated for 25(OH)D values that were less than roughly 38 nmol/L. Another recent large study that supported the role of vitamin D supplementation in reducing the risk of CVD events was conducted on 20,025 vitamin D-deficient subjects wherein treatment to >75 nmol/L levels post supplementation were associated with significantly lower risk of myocardial infraction [48]. An argument, in contrast to pharmaceutical trials, where in nutrient trials like this one, everyone recruited has some of that nutrient in their system, so that fact alone represents a major confounding factor, that has been supported by some earlier studies and lends credence to the single-arm interventional study design for nutrient supplements [49].

Although not part of the objective, it is worthy to note that the ASCVD risk score in the cohort was 6%, with males having a significantly higher risk score than females (9% versus 3%; *p* < 0.001). This score, while not reflective of the general population, nevertheless provides a gross estimate of the prevailing ASCVD risk score among vitamin D-deficient Arab adults. The 10-year ASCVD risk score in Arab males with vitamin D deficiency is much higher compared to Chinese adult males (5%) while the score in Chinese adult females is comparable to Arab adult women with vitamin D deficiency (3%) [50]. Furthermore, among US non-Hispanic White and African American adults aged 40–79 years, more than 25% had greater than a 10% risk (intermediate) of contracting ASCVD [51], a number still considered lower among Arab adults in the present study (36%). The high ASCVD risk score among Arab adults with vitamin D deficiency compliments various epidemiologic data that CVD alone accounts to more than 45% of deaths in Saudi Arabia and this is primarily attributed to low physical activity, a high prevalence of obesity, dyslipidemia, and diabetes [52].

Finally, the relevance of decreasing ASCVD risk score through an increase in HDL cholesterol needs to be considered in light of emerging evidence on the actual protective effects of HDL cholesterol in ASCVD [53]. While no doubt an increase in HDL decreases coronary heart disease by as much as 3% in women and 2% in men independent of known comorbidities [54], this cardioprotective effect appears to follow a U-shaped association where extreme lows and highs in HDL increases mortality for CVD across all age groups but more so for the younger adults, a significant observation obtained from a prospective cohort of 15.8 million Korean adults followed-up for almost 9 years [55]. Regardless, the subsequent increase in HDL cholesterol in the present study following vitamin D status correction, are substantial yet relatively modest in clinical terms, indicating that this subtle increase in HDL cholesterol is enough to alter ASCVD risk scores favorably over time.

The authors acknowledge several limitations. The lack of control group gives it the principal disadvantage of having potential biases from confounding factors. It should be pointed out however that ethical approval would be unobtainable if a placebo group was provided given that all participants have known vitamin D deficiency. Another limitation is the lack of baseline information on diet and physical activity, factors that on their own can improve the cardiometabolic profile of participants. Lastly, the lack of significance in the percentage changes of 10-year ASCVD reduction among those who achieved the optimum vitamin D status maybe due to the small sample size. Despite the limitations, the study is well powered and the first in this homogenous ethnic group to observe the modest benefits of vitamin D correction in terms of reducing the ASCVD score among Arab adults with vitamin D deficiency. The improvement in ASCVD risk score is driven by increased HDL cholesterol.

## 5. Conclusions

In conclusion, vitamin D replenishment confers substantial cardioprotective effects among vitamin D-deficient Arab adults, and slightly more in males, in terms of reducing ASCVD risk score through increasing HDL cholesterol. It is important to consider the present findings with caution, since changes in ASCVD risk score following vitamin D supplementation are not the same as predicted or actual ASCVD, the latter of which needs to be investigated prospectively on a longer term. Nevertheless, the study supports, at least for the vitamin D-deficient adult Arab population, the general recommendation of attempting to achieve optimal vitamin D levels of at least 75 nmol/L or 30 ng/mL and above to support well-being, improved cardiovascular health, and reduced all-cause mortality.

## Figures and Tables

**Table 1:** Baseline characteristics of study participants [N (%)].

Parameters	Total (N = 120)	Males (N = 58)	Females (N = 62)	*p*
BMI [N (%)]				
Lean Overweight Obese NA	15 (12) 59 (49) 43 (36) 3 (2)	7 (12) 34 (59) 16 (28) 1 (2)	8 (13) 25 (40) 27 (44) 2 (3)	0.12
25 (OH) D Status [N (%)]				
Severely deficient (<25 nmol/L) Deficient (25–50 nmol/L)	32 (27) 88 (73)	13 (22) 45 (78)	19 (31) 43 (69)	0.31
Diabetes [N (%)]				
Yes No	27 (22) 93 (78)	12 (20.7) 46 (79)	15 (24) 47 (76)	0.65
Family History of Diabetes [N (%)]				
Yes No NA	66 (55) 17 (14) 37 (31)	23 (40) 4 (7) 31 (53)	43 (69) 13 (21) 6 (10)	<0.001
Family History of CVD [N (%)]				
Yes No NA	15 (12) 62 (52) 43 (36)	5 (9) 17 (29) 36 (62)	10 (16) 45 (73) 7 (11)	<0.001
Family History of Dyslipidemia [N (%)]				
Yes No NA	28 (23) 65 (54) 27 (22)	16 (28) 19 (33) 23 (40)	12 (19) 46 (74) 4 (6)	<0.001
Age (years) #	40.6 ± 10.8	42.8 ± 9.6	38.5 ± 11.6	0.03
Anthropometrics				
Weight (kg) #	77 ± 14	83 ± 14	72 ± 13	<0.01
BMI (kg/m^2^) #	29 ± 5	29 ± 4	30 ± 6	0.36
Waist (cm) #	96 ± 13	98 ± 12	95 ± 14	0.19
Hips (cm) #	102 ± 14	99 ± 13	105 ± 14	0.04
Systolic BP (mmHg) #	127 ± 14	132 ± 10	121 ± 15	<0.01
Diastolic BP (mmHg) #	79 ± 9	83 ± 7	76 ± 10	<0.01
Biochemical Characteristics				
Total cholesterol (mmol/L) #	5.1 ± 1.2	5.1 ± 1.2	5.1 ± 1.2	0.77
Triglycerides (mmol/L) $	1.5 (1.1,2.3)	1.5 (1.1,2.5)	1.5 (1.1,2.1)	0.24
HDL cholesterol (mmol/L) #	1.01 ± 0.4	0.98 ± 0.3	1.03 ± 0.4	0.39
LDL cholesterol (mmol/L) #	3.2 ± 1.1	3.1 ± 1.1	3.3 ± 1.1	0.26
Glucose (mmol/L) $	5.7 (5.1,6.6)	5.7 (5.3,6.6)	5.6 (4.9,6.5)	0.35
Total 25 (OH)D (nmol/L) #	32.5 ± 10.7	34.7 ± 9.5	30.4 ± 11.5	0.03
Category by Cardiovascular Risk Score (ASCVD) [N (%)]				
Low Borderline Intermediate High	49 (41) 22 (18) 43 (36) 6 (5)	42 (68) 4 (6) 16 (26) 0 (0)	7 (12) 18 (31) 27 (47) 6 (10)	<0.001
ASCVD risk score (%) $	6 (2,10)	9 (6,13)	3 (1, 8)	<0.001

Note: Data presented as N (%) for categorical variables; mean ± SD for continuous normal variables (#); and medians (25th percentile, 75th percentile) for continuous non-normal variables ($). The difference between variables in both sexes was calculated by independent sample *t*-tests and Mann–Whitney U-tests for Gaussian and non-Gaussian variables, respectively, and χ^2^ test for categorical variables. *p* < 0.05 is considered significant. NA: data not available; BP: blood pressure; HDL: high density lipoprotein; VDBP: vitamin D binding protein.

**Table 2:** Changes in biochemical characteristics of study participants post intervention.

Parameters	All (N = 120)			Males (N = 58)			Females (N = 62)		
	Follow-Up	Difference	*p*-Value	Follow-Up	Difference	*p*-Value	Follow-Up	Difference	*p*-Value
Total cholesterol (mmol/L) #	5.1 ± 1.3	−0.02	0.83	5.2 ± 1.4	0.1	0.46	5.0 ± 1.2	−0.1	0.31
Triglycerides (mmol/L) $	1.6 (1.1,2.1)	0.1	0.82	1.7 (1.3,2.3)	0.2	0.60	1.4 (1,2)	−0.1	0.40
HDL cholesterol (mmol/L) #	1.1 ± 0.4	0.1	0.001	1.0 ± 0.3	0.1	0.001	1.2 ± 0.4	0.2	0.04
LDL cholesterol (mmol/L) #	3.1 ± 1.1	−0.1	0.28	3.2 ± 1.3	0.1	0.65	3.1 ± 1	−0.2	0.04
Glucose (mmol/L) $	5.8 (5.2,6.4)	0.1	0.62	5.9 (5.5,6.4)	0.2	0.24	5.5 (4.8,6.4)	−0.1	0.74
Total 25 (OH)D (nmol/L) #	63.3 ± 16.5	30.8	<0.001	61 ± 14.3	26.2	<0.001	65.5 ± 18.1	35.1	<0.001
ASCVD risk score (%) $	5.0 (1.5,8.3)	−1.1	<0.001	7.4 (5.4,12)	−1.4	0.02	1.5 (0.8,3.5)	−1.1	<0.001

Note: Data presented as mean ± SD for continuous normal variables (#); and medians (25th percentile, 75th percentile) for continuous non-normal variables ($). The difference between variables post intervention versus baseline was calculated by related samples *t*-test and Wilcoxon matched-pair signed rank test for Gaussian and non-Gaussian variables, respectively. *p* < 0.05 is considered significant.

**Table 3:** Percentage changes in ASCVD risk and components according to post-supplementation 25(OH)D status.

Post-Intervention Status	25(OH)D < 75 nmol/L		25(OH)D ≥ 75 nmol/L		*p*-Value *
	% Change	*p*-Value	% Change	*p*-Value	
All Participants	105		25		
25(OH)D (nmol/L)	76	<0.001	159	<0.001	<0.001
Total cholesterol (mmol/L)	−0.2	0.95	−2	0.71	0.54
Triglycerides (mmol/L)	12	0.98	−2	0.58	0.06
HDL cholesterol (mmol/L)	11	0.01	12	0.009	0.16
LDL cholesterol (mmol/L)	−3	0.35	−3	0.62	0.86
Glucose (mmol/L)	3	0.56	−2	0.83	0.79
10-year ASCVD risk (%)	−18	0.01	−23	0.03	0.41
Males	49		9		
25(OH)D (nmol/L)	62	<0.001	149	<0.001	0.002
Total cholesterol (mmol/L)	3	0.39	−0.4	0.95	0.97
Triglycerides (mmol/L)	7	0.83	3	0.44	0.36
HDL cholesterol (mmol/L)	6	0.02	8	0.02	0.46
LDL cholesterol (mmol/L)	3	0.51	−3	0.85	0.56
Glucose (mmol/L)	4	0.57	8.4	0.13	0.35
10-year ASCVD risk (%)	−10	0.02	−26	<0.001	0.71
Females	46		16		
25(OH)D (nmol/L)	95	<0.001	166	<0.001	<0.001
Total cholesterol (mmol/L)	−3	0.38	−2	0.59	0.49
Triglycerides (mmol/L)	2	0.73	−7	0.31	0.07
HDL cholesterol (mmol/L)	16	0.03	15	0.03	0.38
LDL cholesterol (mmol/L)	−8	0.05	−3	0.51	0.79
Glucose (mmol/L)	−2	0.83	−9	0.22	0.32
10-year ASCVD risk (%)	−32	0.004	−47	0.002	0.69

Note: Data presented as % change calculated by difference in mean or median levels/baseline mean or median level * 100. *p*-value * represents differences in percent changes in each variable in the two vitamin D improvement groups. *p* < 0.05 is considered significant.

## Data Availability

The data presented in this study are available on request from the corresponding author.

## Supplementary Materials

The following supporting information can be downloaded at: https://www.mdpi.com/article/10.3390/nu15030551/s1, Table S1: Change in lipid indices and ASCVD risk score according to 25(OH) vitamin D improvement tertiles. Figure S1: ASCVD Risk Estimator.
