# Supplementary material for: Improved HDL Cholesterol through Vitamin D Status Correction Substantially Lowers 10-Year Atherosclerotic Cardiovascular Disease Risk Score in Vitamin D-Deficient Arab Adults
Source: Nutrients. 2023 Jan 20;15(3):551. doi: 10.3390/nu15030551 (PMC9921125; doi:10.3390/nu15030551)
Supplement: Supplementary file 1 [file nutrients-15-00551-s001.zip › nutrients-2073690-supplementary.pdf]

Supplementary Figure S1. ASCVD Risk Estimator

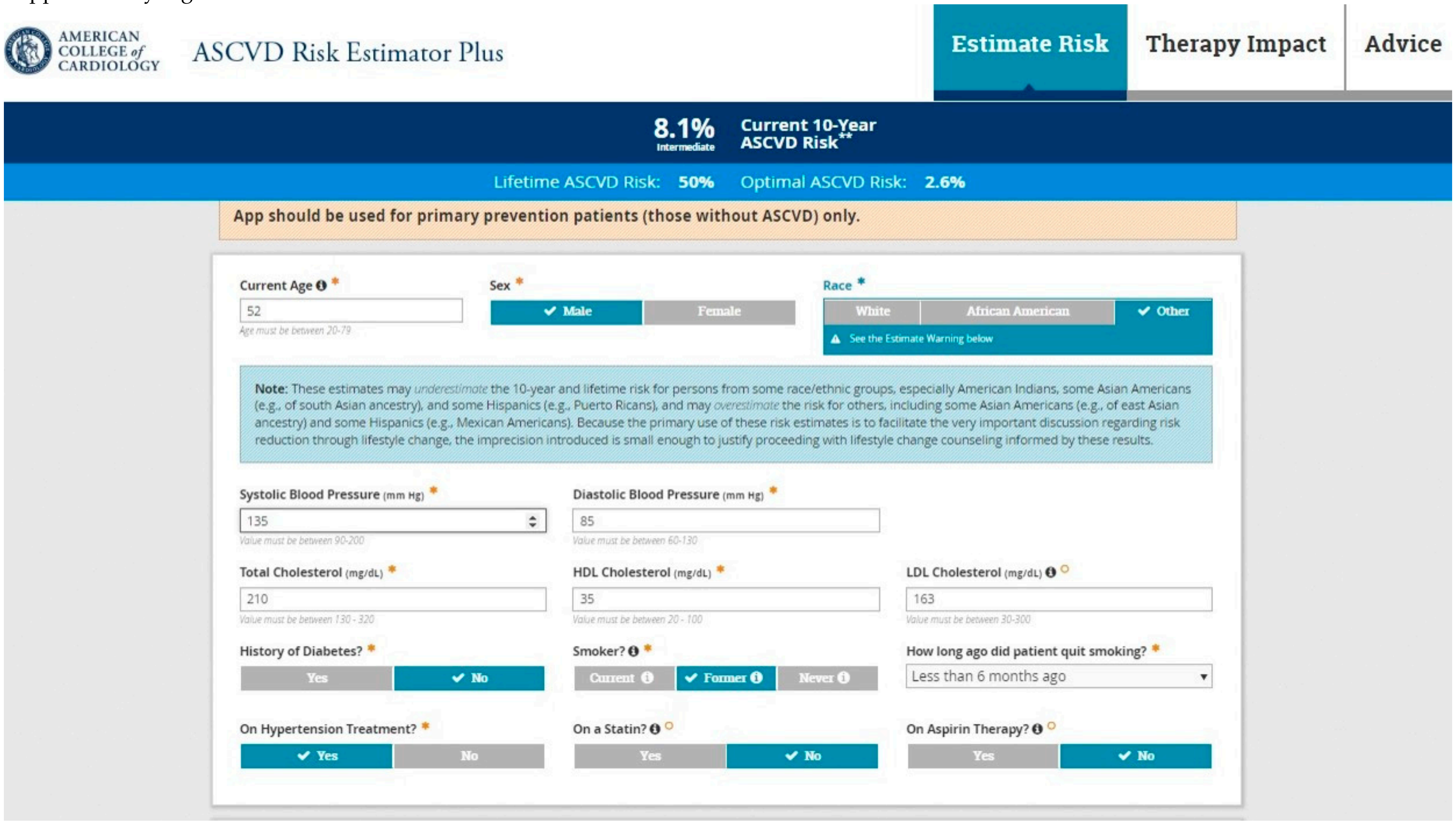

Supplementary Table S1: Changes in Lipid indices and ASCVD risk score according to 25(OH) vitamin D improvement tertiles.

	Baseline	End of study	% change	Baseline	End of study	% change	Baseline	End of study	% change	p^{t}
**Parameters**	**Tertile 1**			**Tertile 2**			**Tertile 3**			
**All Subjects**										
Total 25 (OH) Vit D	38.3 ± 7.1	54.0 ± 6.1	41	32.8 ± 11.1	58.5 ± 11.2	78	26.4 ± 10.1	77.4 ± 18.6	193	<0.01
Total Cholesterol	4.9 ± 1	4.8 ± 1.1	-3	5.3 ± 1.4	5.4 ± 1.5	2	5.0 ± 1.2	5.1 ± 1.2	0.2	0.10
Triglycerides (mmol/l)$	1.8	1.7 (1.3,2.3)	-4	1.5 (1.2,2.4)	1.7 (1,2.1)	10	1.4 (0.9,2)	1.4 (1.1,2)	1	0.30
HDL-Cholesterol	1 ± 0.4	1.1 ± 0.3	4	0.96 ± 0.4	1.11 ± 0.5	16	1.03 ± 0.4	1.19 ± 0.4	16	0.03
LDL-Cholesterol	2.9 ± 1	2.8 ± 1.1	-4	3.4 ± 1.3	3.4 ± 1.3	-0.3	3.3 ± 1	3.2 ± 1	-4	0.05
Glucose (mmol/l)$	5.6	5.8 (5.2,7)	3	5.6 (5.2,6.3)	5.6 (5.1,6)	0.2	6.0 (5.1,6.4)	5.8 (5.1,6.5)	-3	0.67
ASCVD risk score (%) $	6.3	5.8 (3.5,8.6)	-8	7.3	5.6	-24	4.2 (1.5,8.7)	2.4 (0.8,6)	-44	0.02
**Males**										
Total 25 (OH) Vit D	39.7 ± 5.5	54.0 ± 4.7	36	33.5 ± 10.9	57.0 ± 10.5	70	30.7 ± 9.3	72.3 ± 17.5	135	<0.01
Total Cholesterol	4.6 ± 0.9	4.8 ± 1.3	4	5.0 ± 1.3	5.2 ± 1.2	3	5.7 ± 1.3	5.6 ± 1.6	-1	0.19
Triglycerides (mmol/l)$	1.6	1.9 (1.4,2.3)	15	1.5 (1,2.4)	1.6 (1,2)	12	1.9 (1.3,2.6)	1.6 (1.2,2.6)	-18	0.55
HDL-Cholesterol	1 ± 0.3	1 ± 0.3	2	0.87 ± 0.4	0.97 ± 0.3	12	1.06 ± 0.3	1.15 ± 0.3	8	0.04
LDL-Cholesterol	2.7 ± 0.9	2.7 ± 1.1	1	3.0 ± 1	3.2 ± 1.2	7	3.7 ± 1.2	3.6 ± 1.5	-2	0.10
Glucose (mmol/l)$	5.7	5.8 (5.4,6.8)	3	5.7 (5.2,6.4)	5.8 (5.6,6.1)	2	5.9 (5.4,6.4)	5.9 (5.5,6.6)	1	0.79
ASCVD risk score (%) $	7.2 (5,9.7)	7.5	4	9.9	6.6	-33	9.9	7.7	-22	0.02
**Females**										
Total 25 (OH) Vit D	37.1 ± 7.8	54.8 ± 7.5	48	28.9 ± 13.2	58.4 ± 13.2	102	25.7 ± 10.2	81.6 ± 17.7	217	<0.01
Total Cholesterol	5.1 ± 1	4.7 ± 0.9	-8	5.5 ± 1.5	5.4 ± 1.5	-2	4.8 ± 1.2	4.9 ± 1.1	2	0.13
Triglycerides (mmol/l)$	2.0	1.5 (1.1,2.3)	-26	1.4 (1.2,1.8)	1.7 (1,2.3)	24	1.4 (0.8,2.1)	1.3 (0.9,1.8)	-3	0.59
HDL-Cholesterol	0.96 ± 0.5	1.04 ± 0.3	8	1.18 ± 0.4	1.27 ± 0.5	8	0.97 ± 0.3	1.28 ± 0.4	32	0.02
LDL-Cholesterol	3.2 ± 0.8	2.9 ± 0.9	-8	3.7 ± 1.3	3.3 ± 1.3	-9	3.2 ± 0.9	3.0 ± 0.7	-5	0.38
Glucose (mmol/l)$	5.8	5.8 (5.2,7.1)	1	5.5 (4.9,6.2)	5.4 (4.6,6)	-2	6.0 (4.9,6.4)	5.5 (4.6,6.3)	-8	0.14
ASCVD risk score (%) $	2.9	1.5 (0.8,5.6)	-48	1.6 (0.9,3.6)	1.45	-9	4.05	1.3 (0.8,4.6)	-68	0.27

Note: Data presented Mean±SD for continuous normal variables (#); and medians (25th percentile, 75th percentile) for continuous non-normal variables ($). % change is calculated by difference in mean or median levels/ baseline mean or median level *100. P^t represents differences (trend) in percent changes in each variable in the three vitamin D improvement tertiles and was calculated by ANOVA. $p<0.05$ is taken as significant.
